# Severe Neonatal Infection in Crisponi Syndrome: A Diagnostic Challenge in a Preterm Infant

**DOI:** 10.7759/cureus.108647

**Published:** 2026-05-11

**Authors:** Divya Ravikumar, Garayeva Sabina

**Affiliations:** 1 Department of Paediatrics, Azerbaijan Medical University, Baku, AZE

**Keywords:** autonomic dysfunction, bilateral pneumonia, crisponi syndrome, crlf1 mutation, neonatal sepsis, prematurity, stimulus-induced spasms

## Abstract

Crisponi syndrome, also referred to as cold-induced sweating syndrome type 1, is an uncommon autosomal recessive disorder caused by mutations in the cytokine receptor-like factor 1 (*CRLF1*) gene. The disorder typically presents with distinctive craniofacial abnormalities, feeding impairment, stimulus-induced muscle spasms, and varying degrees of autonomic dysfunction. Neonates affected by this condition are particularly vulnerable to respiratory complications and infections, especially in the setting of prematurity.

We report a case of a preterm male neonate born at 34 weeks of gestation to consanguineous parents, presenting with characteristic dysmorphic features and early-onset stimulus-induced spastic episodes. Initially suspected to have hypoxic-ischemic encephalopathy, the patient later developed severe respiratory distress and sepsis secondary to bilateral pneumonia. Laboratory findings showed progressive inflammatory markers, hematological abnormalities, and a positive blood culture for *Enterobacter* species. Retrospective assessment using the neonatal Sequential Organ Failure Assessment score yielded a score of 11, indicating severe organ dysfunction and high mortality risk. Whole exome sequencing confirmed a homozygous likely pathogenic variant in the *CRLF1* gene, consistent with Crisponi syndrome.

Despite intensive care management, the patient succumbed to multiorgan failure. This case highlights the diagnostic challenges in distinguishing neurological manifestations of Crisponi syndrome from hypoxic brain injury and underscores the high risk of severe infection in affected preterm neonates. Early genetic diagnosis and multidisciplinary management are critical for improving outcomes.

## Introduction

Crisponi syndrome is a rare autosomal recessive disorder caused by mutations in the cytokine receptor-like factor 1 (*CRLF1*) gene and is classified within the spectrum of cold-induced sweating syndromes [1-3]. It is characterized by distinctive craniofacial dysmorphism, feeding difficulties, episodic stimulus-induced muscle spasms, and autonomic dysfunction, including temperature instability [1,2]. Since its first description, fewer than 100 cases have been reported in the literature, highlighting its rarity and the limited clinical experience available for guiding diagnosis and management [1].

The* CRLF1* gene encodes cytokine receptor-like factor 1, which forms a heterodimeric complex with cardiotrophin-like cytokine factor 1 (CLCF1) and signals through the ciliary neurotrophic factor receptor (CNTFR) pathway [1]. This signaling pathway plays a critical role in neuronal survival, autonomic regulation, and motor neuron development. Disruption of this pathway leads to impaired neurotrophic signaling, which underlies the characteristic autonomic instability, abnormal motor responses, and feeding dysfunction observed in Crisponi syndrome [2].

The neonatal period is particularly critical, as affected infants are at high risk of life-threatening complications, including feeding impairment, recurrent spasms, and respiratory compromise [2]. Mortality in early infancy remains significant, often related to autonomic instability, aspiration, and respiratory failure [2]. These risks may be further exacerbated in preterm neonates due to immunological immaturity, reduced physiological reserves, and increased susceptibility to severe infections [4].

Early manifestations of Crisponi syndrome can closely mimic hypoxic-ischemic encephalopathy (HIE), particularly in the presence of abnormal tone, feeding difficulties, and seizure-like activity [1,3]. This overlap can lead to initial misdiagnosis and delay in appropriate genetic evaluation. Furthermore, impaired swallowing and autonomic dysfunction predispose affected neonates to aspiration and subsequent respiratory infections, increasing the likelihood of severe sepsis [2,4].

Despite these known features, there is limited literature describing the interaction between prematurity, Crisponi syndrome, and severe neonatal sepsis, particularly in cases complicated by culture-proven infections and multiorgan failure. The coexistence of these factors presents a complex diagnostic and management challenge with significant implications for clinical outcomes.

In this context, early recognition is critical yet challenging, particularly in preterm neonates, where overlapping clinical features may obscure the diagnosis. We present a clinically complex case of a preterm neonate with genetically confirmed Crisponi syndrome complicated by severe bilateral pneumonia and gram-negative sepsis, ultimately resulting in a fatal outcome. This case aims to highlight the diagnostic challenges, clinical course, and importance of early recognition and multidisciplinary management in such high-risk neonatal populations.

## Case presentation

A male infant was born at 34 weeks of gestation via spontaneous vaginal delivery to a 27-year-old mother in a third-degree consanguineous marriage. This was the third pregnancy and third delivery of the mother. The neonate remained admitted in the neonatal intensive care unit from birth for supportive care and monitoring.

The birth weight was 2300 g. Apgar scores were 7 at 1 minute and 8 at 5 minutes. At birth, the neonate was noted to have multiple dysmorphic features suggestive of an underlying congenital syndrome. These included a round facial appearance, a depressed nasal bridge, anteverted nostrils, and low-set ears with simplified auricular folds. Additional craniofacial abnormalities included a small mouth with relative macroglossia, a long philtrum, and micrognathia. Examination of the extremities revealed bilateral camptodactyly and persistently clenched fists, further supporting the presence of a syndromic phenotype.

Within the first few hours of life, the patient developed recurrent paroxysmal episodes characterized by marked hypertonia, opisthotonic posturing, and generalized tremors. These episodes were accompanied by involuntary contractions of the facial and oropharyngeal musculature. Notably, the events were consistently triggered by external stimuli, including crying and painful procedures, and demonstrated a reproducible pattern. Each episode subsided following removal of the precipitating stimulus and administration of supplemental oxygen, suggesting a non-epileptic, stimulus-sensitive mechanism.

Given the severity of the initial clinical presentation, including respiratory compromise and neurological manifestations, the neonate required immediate supportive care. Admission to a neonatal incubator for thermoregulation and close monitoring, and initiation of oxygen therapy were necessary to maintain adequate oxygenation.

Following an initial period of partial stabilization after birth, the patient’s clinical condition progressively deteriorated, necessitating re-hospitalization. At 1 month and 20 days of age, he was admitted in a critical state.

On admission, the patient exhibited severe respiratory distress characterized by marked tachypnea and increased work of breathing. Cardiovascular assessment revealed disturbances in cardiac rhythm, indicating hemodynamic instability. Neurologically, the infant was lethargic and demonstrated a complete absence of the swallowing reflex, significantly impairing feeding ability. Recurrent seizure-like spastic episodes (non-epileptic, stimulus-induced tonic spasms characterized by hypertonia and opisthotonic posturing) were also observed. Electroencephalography (EEG) showed no epileptiform discharges, supporting a non-epileptic origin of the spasms. At this stage, the constellation of neurological findings, including impaired swallowing and seizure-like activity, was initially interpreted as secondary to hypoxic injury of the central nervous system.

Laboratory evaluation during the hospital course revealed a progressive inflammatory and hematological deterioration consistent with a severe systemic infectious process. The patient demonstrated marked leukocytosis, with an initial white blood cell count of 24.62 × 10³/µL. Hemoglobin levels showed a progressive decline from 9.5 g/dL to 7.8 g/dL, indicating worsening anemia. Thrombocytopenia was also observed, with a platelet count of 94 × 10³/µL.

As shown in Table 1, inflammatory markers were significantly elevated, with C-reactive protein (CRP) levels showing a progressive rise from 47 mg/L to 144 mg/L. Procalcitonin was elevated at 1.02 ng/mL, further supporting a bacterial infectious process. Liver enzyme analysis revealed elevated aspartate aminotransferase (AST) levels reaching 124.7 U/L, while alanine aminotransferase (ALT) remained relatively within normal limits, suggestive of systemic illness-related hepatic stress rather than primary hepatocellular injury. Electrolyte imbalance was also noted during the clinical course, reflecting metabolic instability in the context of critical illness (Table 1).

**Table 1:** Dynamics of laboratory parameters during hospitalization Values outside the reference range are indicative of pathological changes consistent with systemic infection and organ dysfunction. Laboratory values correspond to key clinical phases, including admission, early deterioration, and advanced critical illness. ALT: alanine transaminase; AST: aspartate aminotransferase.

Parameter	27-02-2026	02-03-2026	08-03-2026	14-03-2026	22-03-2026	Reference range
White blood cells (×10³/µL)	24.62	15.04	9.88	13.13	13.13	5-20
Red blood cells (×10⁶/µL)	2.82	2.61	3.03	2.54	2.90	3.5-5.5
Hemoglobin (g/dL)	9.5	8.5	9.4	7.8	8.7	13-20
Hematocrit (%)	27.9	25.4	28.8	24.5	27.3	40-60
Platelets (×10³/µL)	420	235	94	314	272	150-400
C-reactive protein (mg/L)	47.22	75.07	—	138.09	144.62	<5
Procalcitonin (ng/mL)	1.02	—	—	—	—	<0.5
AST (U/L)	38.9	—	—	124.7	—	<40
ALT (U/L)	26.9	—	—	—	—	<40
Potassium (mmol/L)	5.54	—	—	4.48	—	3.5-5.5
Sodium (mmol/L)	140	—	—	135	—	135-145

The progressive rise in CRP and persistent elevation of inflammatory markers, in conjunction with thrombocytopenia and anemia, reflect an evolving systemic inflammatory response consistent with severe neonatal sepsis and impending multiorgan dysfunction collectively indicate progression to septic shock.

Blood cultures were obtained at the time of clinical deterioration (day 1 of admission) before initiation of antibiotic therapy. Microbiological analysis of blood cultures yielded *Enterobacter* species, confirming the presence of a gram-negative bloodstream infection. Radiological investigations demonstrated bilateral pneumonic infiltrates on chest radiographs, with a transient period of partial radiological improvement followed by deterioration. Abdominal radiography revealed intestinal meteorism without evidence of free intraperitoneal air. Ultrasonographic examination showed mild splenomegaly; however, no significant structural abnormalities were identified.

**Figure 1:**
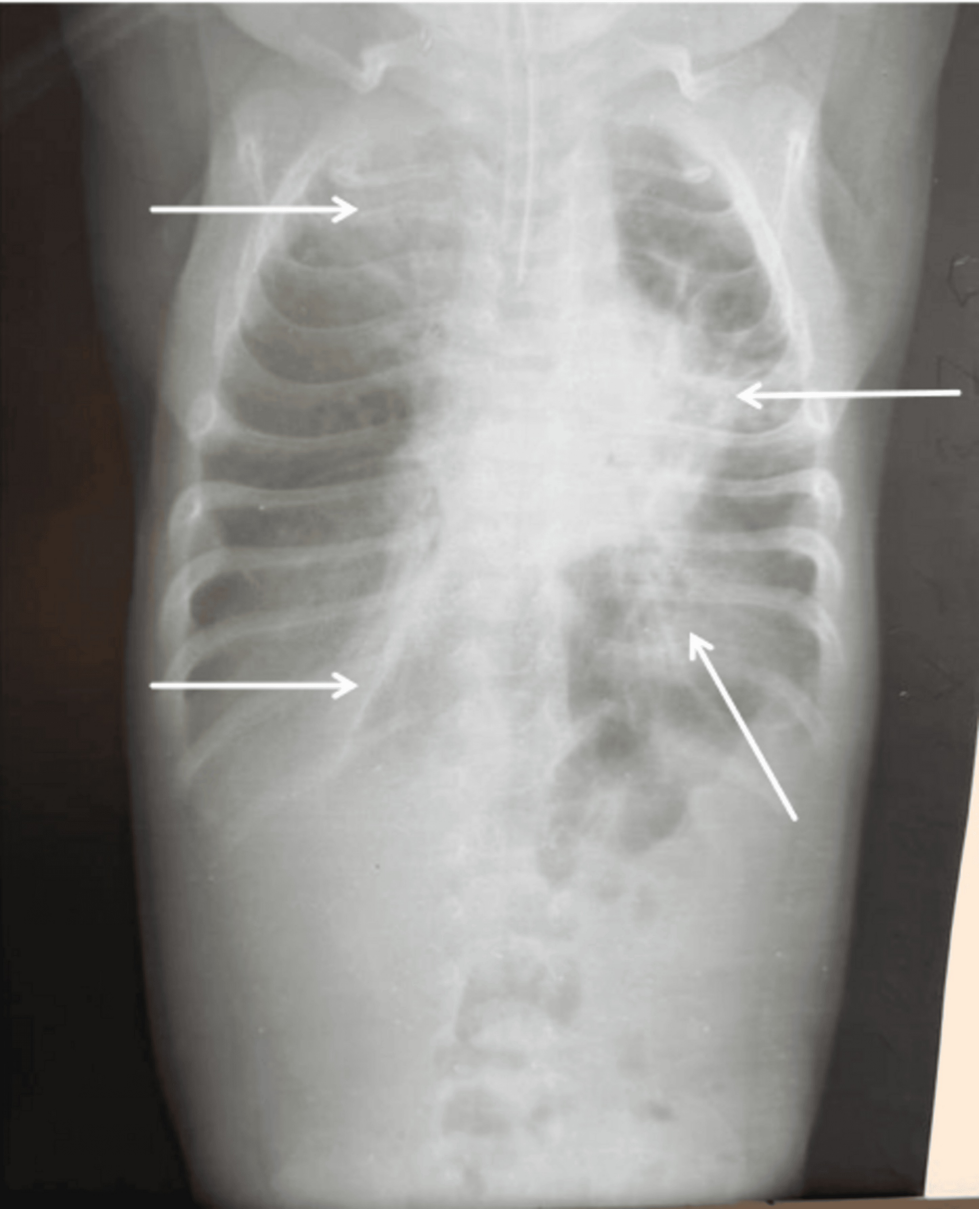
Chest radiograph demonstrating bilateral infiltrates at admission Arrows indicate areas of consolidation consistent with pneumonia.

**Figure 2:**
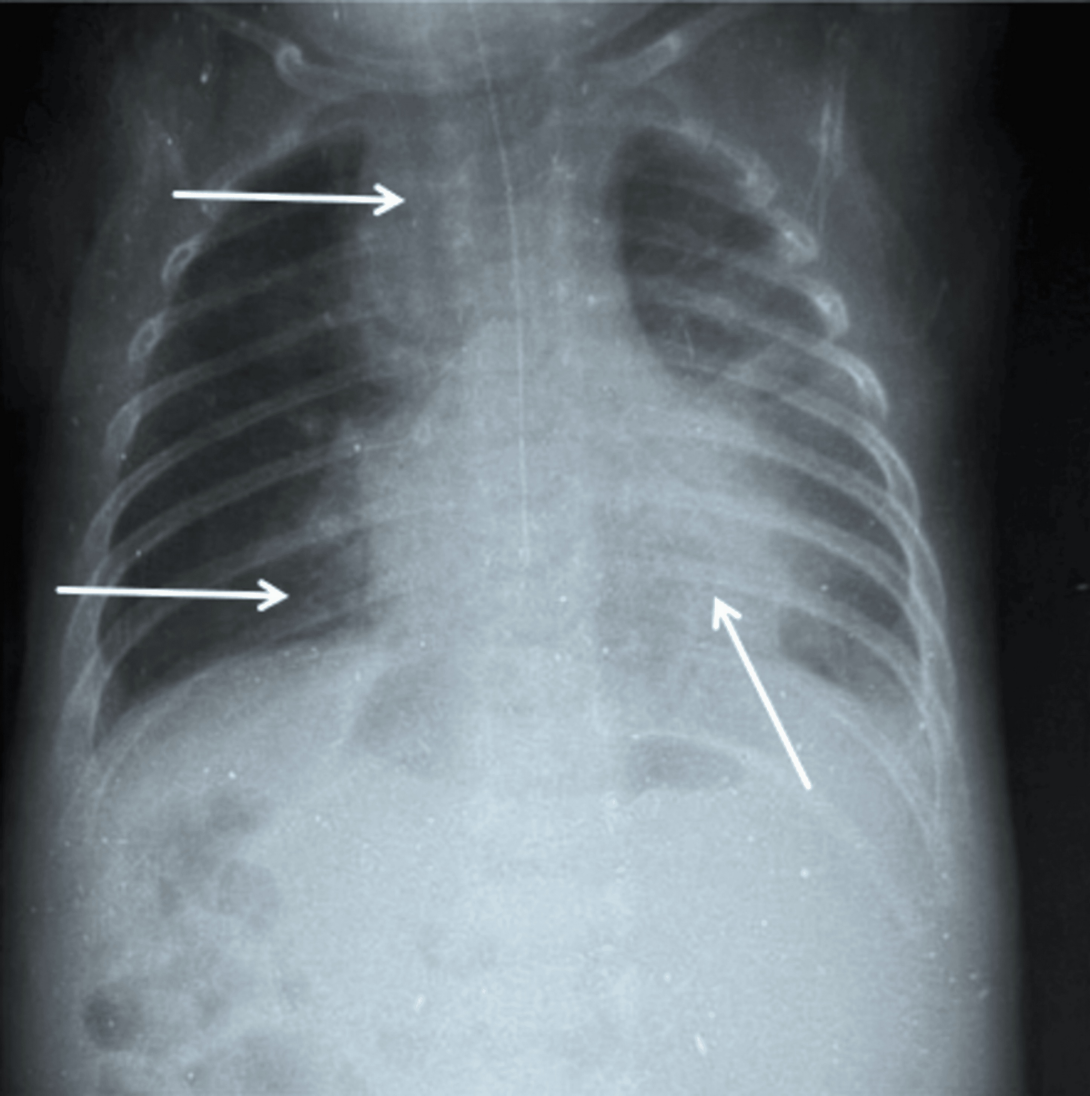
Chest radiograph (day 7) showing progression to pulmonary opacities

**Figure 3:**
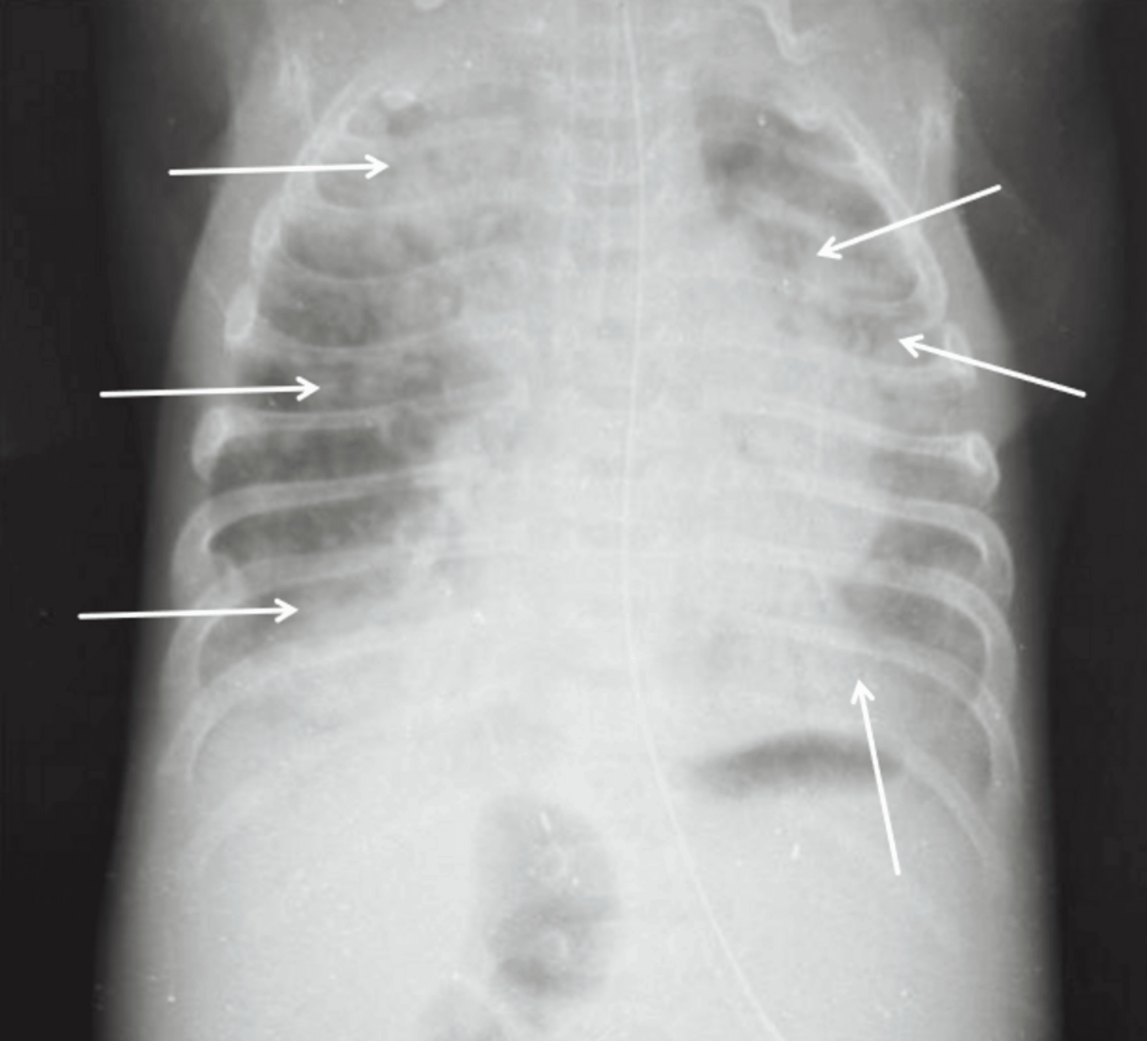
Chest radiograph (day 24) depicting marked deterioration with diffuse bilateral opacification

Serial chest radiographs demonstrated a progressive and ultimately fulminant course, characterized by initial bilateral infiltrates, a progressive period of pulmonary opacities, and subsequent diffuse opacification of both lungs, consistent with end-stage severe pneumonia (Figures 1-3).

Whole-exome sequencing (WES) was performed during the course of hospitalization in view of persistent dysmorphic features and unexplained neurological findings, and identified a homozygous likely pathogenic variant in the *CRLF1* gene, confirming the diagnosis of Crisponi syndrome. 

Based on the clinical presentation, laboratory findings, microbiological results, and genetic analysis, the patient was diagnosed with Crisponi syndrome, as confirmed by the identification of a homozygous likely pathogenic variant in the *CRLF1* gene on WES. In addition, the patient developed bilateral pneumonia, as evidenced by radiological findings of bilateral pulmonary infiltrates. The condition progressed to acute respiratory failure, necessitating advanced respiratory support during the course of hospitalization.

The overall clinical course was further complicated by prematurity-associated complications, which contributed to increased susceptibility to infection and multiorgan dysfunction. The patient was managed in the intensive care unit with a comprehensive multidisciplinary approach. Respiratory support was provided using a stepwise escalation strategy, including synchronized ventilation approaches, synchronized intermittent mandatory ventilation, non-invasive ventilation, and continuous positive airway pressure, depending on the patient’s evolving respiratory status.

Broad-spectrum intravenous antibiotics (amikacin) were administered to address culture-proven neonatal sepsis and severe bilateral pneumonia. Hemodynamic instability was managed with inotropic support, including dopamine and adrenaline, to maintain adequate perfusion and cardiovascular stability. In view of recurrent seizure-like spastic episodes, anticonvulsant therapy was initiated. Strict fluid and electrolyte management was implemented to correct metabolic imbalances and maintain homeostasis during critical illness. In addition, the patient received continuous supportive and symptomatic care throughout the hospital course, including intensive monitoring and organ support measures as clinically indicated.

**Figure 4:**
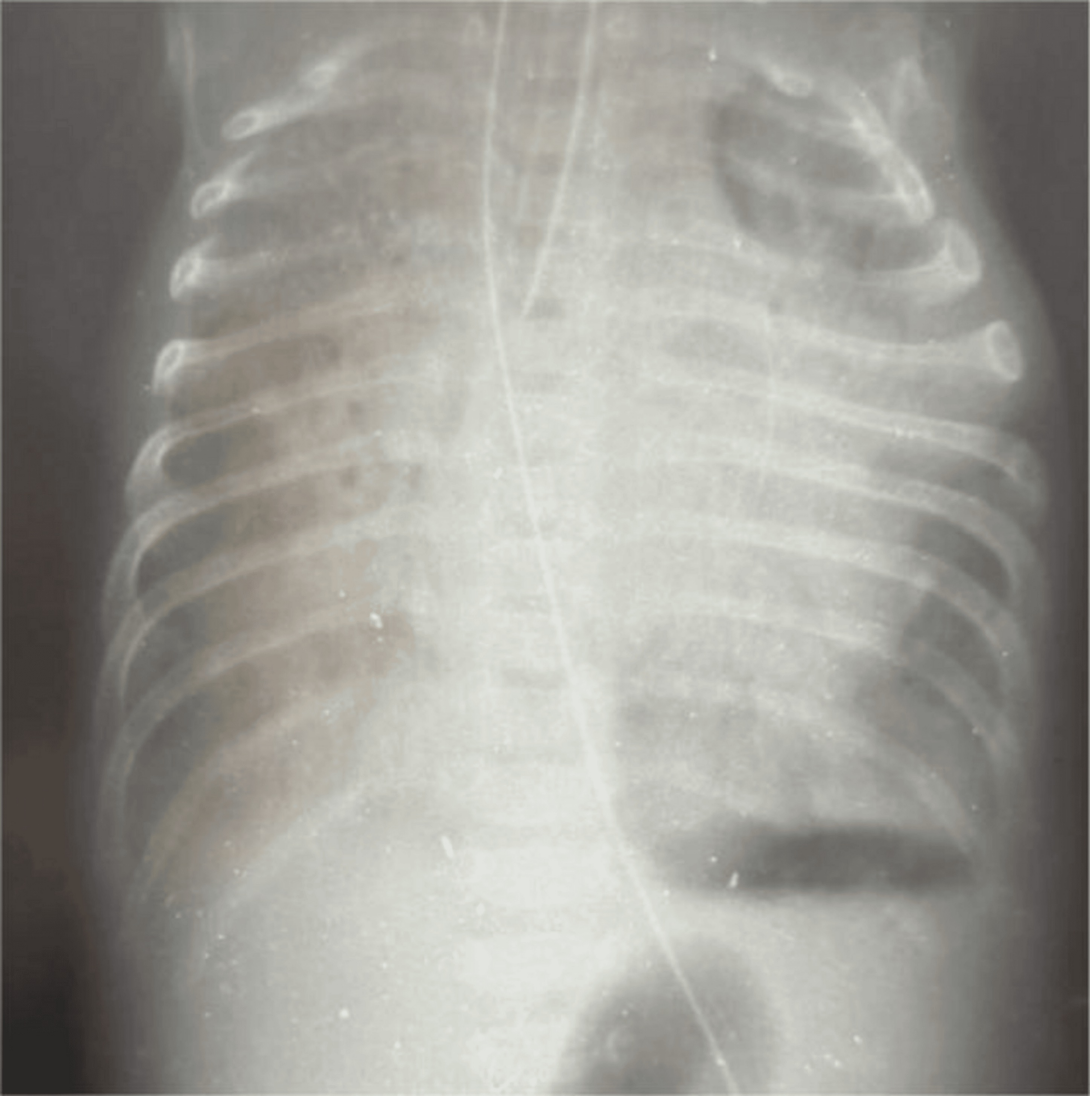
Chest radiograph demonstrating near-complete bilateral lung involvement on the day of death

Despite aggressive management, the patient’s condition progressively worsened. At the age of 2 months and 16 days, he developed cardiac arrest. Resuscitation was unsuccessful, and death was declared (Figure 4).

## Discussion

This case highlights the complex interaction between underlying genetic pathology, prematurity, and severe neonatal infection, all of which contributed to the rapidly progressive and fatal clinical course. Prematurity is a well-established risk factor for severe neonatal morbidity and mortality due to the immaturity of the immune system, reduced transplacental transfer of maternal antibodies, and limited respiratory reserve, which together predispose neonates to rapid decompensation in the setting of infection [4].

Importantly, the patient was a late preterm infant (34 weeks’ gestation) who was large for gestational age at birth (>97th percentile), reflecting the overall perinatal complexity of the case. Postnatal growth remained within the upper normal range, with a body weight of 4000 g at 1 month and 20 days (approximately term-equivalent age), indicating adequate growth for corrected age.

In addition, Crisponi syndrome further increased the patient’s vulnerability to complications. The disorder is characterized by impaired swallowing, which significantly increases the risk of aspiration, as well as autonomic dysfunction and stimulus-induced spastic episodes that may clinically mimic epileptic seizures [1-3]. The characteristic autonomic dysfunction and stimulus-induced spasms in Crisponi syndrome are believed to result from impaired neurotrophic signaling due to *CRLF1* dysfunction. *CRLF1* forms a functional complex with CLCF1, which signals through the CNTFR pathway. This signaling pathway plays a critical role in autonomic regulation, motor neuron development, and neuronal survival. Disruption of CNTF-related pathways affects motor neuron excitability and autonomic regulation, leading to exaggerated responses to external stimuli, abnormal muscle tone, and temperature instability. These features collectively contribute to feeding difficulties, respiratory compromise, and diagnostic confusion in the early neonatal period.

A major diagnostic challenge in this case was the initial misinterpretation of neurological manifestations as HIE. The differential diagnoses considered in this case, along with their distinguishing clinical features and diagnostic considerations, are summarized in Table 2, highlighting the key factors that supported the diagnosis of Crisponi syndrome over other conditions. However, the observed episodes were later recognized as consistent with Crisponi syndrome, in which spasms are typically stimulus-induced, transient, and non-epileptic in nature [1,3].

**Table 2:** Differential diagnosis with clinical correlation *CRLF1*: cytokine receptor-like factor 1; CRP: C-reactive protein; EEG: electroencephalogram; MRI: magnetic resonance imaging.

Condition	Key clinical features	Findings supporting the diagnosis in this case	Findings against diagnosis/exclusion	Diagnostic tests
Hypoxic-ischemic encephalopathy	Perinatal asphyxia, seizures, poor tone, and feeding difficulty	Absence of swallowing reflex, seizure-like activity, and lethargy	No clear history of birth asphyxia; stimulus-induced spasms atypical; persistent dysmorphism unexplained	MRI brain, cord blood gases, and clinical history
Neonatal sepsis	Respiratory distress, lethargy, temperature instability, and shock	Elevated CRP and procalcitonin, leukocytosis, thrombocytopenia, positive blood culture (*Enterobacter*), and pneumonia	Does not explain congenital dysmorphic features or early stimulus-induced spasms	Blood culture, CRP, and procalcitonin
Neonatal tetanus	Generalized rigidity, spasms triggered by stimuli, and feeding difficulty	Stimulus-induced spasms and opisthotonus	Typically presents after the first week; no umbilical infection; maternal immunization history not suggestive; rare in hospital delivery	Clinical diagnosis and history
Epileptic encephalopathy	Refractory seizures and developmental delay	Seizure-like episodes and abnormal tone	Episodes triggered by stimuli and relieved by removal are more consistent with non-epileptic spasms; no EEG confirmation	EEG and neuroimaging
Pierre Robin sequence	Micrognathia, glossoptosis, and airway obstruction	Micrognathia and feeding difficulty	Does not explain spasms, autonomic dysfunction, or systemic features	Clinical examination
Trisomy 21	Hypotonia, characteristic facies, and congenital anomalies	Dysmorphic features and macroglossia	Absence of typical hypotonia; presence of spasms and autonomic dysfunction not typical	Karyotyping
Congenital myopathy	Hypotonia, weakness, and feeding difficulty	Feeding difficulty and respiratory distress	Hypertonic spasms rather than hypotonia; stimulus-induced episodes atypical	Muscle biopsy and genetic testing
Inborn errors of metabolism	Lethargy, seizures, and metabolic acidosis	Neurological symptoms and deterioration	No metabolic acidosis or hypoglycemia reported; dysmorphic features less typical	Metabolic panel, ammonia, and lactate
Crisponi syndrome	Dysmorphism, stimulus-induced spasms, feeding difficulty, and autonomic dysfunction	Classic facial features, camptodactyly, stimulus-induced spasms, absent swallowing reflex, and consanguinity	None after genetic confirmation	Whole-exome sequencing (*CRLF1* mutation)

The diagnosis of sepsis was strongly supported by a combination of clinical, laboratory, and microbiological evidence, including markedly elevated inflammatory markers such as CRP and procalcitonin, positive blood cultures growing *Enterobacter* species, progressive clinical deterioration, and the presence of bilateral pneumonia as a clear infectious focus. Elevated AST levels in the presence of relatively normal ALT values were interpreted as reflecting systemic inflammation and tissue injury related to severe critical illness, rather than primary hepatic pathology. Gram-negative infections are known to be associated with severe disease and high mortality in neonates [4].

The observed radiological progression further supports the severe and rapidly evolving infectious process, consistent with a fulminant course of bilateral pneumonia culminating in respiratory failure.

The neonatal Sequential Organ Failure Assessment (nSOFA) score was retrospectively applied to quantify organ dysfunction and assess mortality risk in this patient. The patient’s nSOFA score of 11/15 indicates severe multiorgan dysfunction and is strongly associated with a high risk of mortality, reflecting advanced physiological compromise involving multiple organ systems. The high nSOFA score was driven primarily by severe respiratory failure requiring ventilatory support, cardiovascular instability necessitating vasoactive agents, and hematologic involvement in the form of thrombocytopenia (Table 3).

**Table 3:** Retrospective nSOFA scoring in the present case CPAP: continuous positive airway pressure; NIV: non-invasive ventilation; nSOFA: neonatal Sequential Organ Failure Assessment; SIMV: synchronized intermittent mandatory ventilation.

Component	Clinical findings in this patient	Score
Respiratory	Requirement for invasive and non-invasive ventilation (SIMV, NIV, and CPAP); severe respiratory failure with bilateral pneumonia	6
Cardiovascular	Requirement for vasoactive support (dopamine and adrenaline), indicating significant hemodynamic instability	4
Hematologic (platelets)	Platelets: 94 × 10³/µL	1
Total nSOFA score	—	11

Recent literature strongly supports the prognostic utility of the nSOFA score across diverse neonatal populations. A systematic review demonstrated that nSOFA has good-to-excellent mortality discrimination, with reported area under the receiver operating characteristic curve (AUROC) values ranging from ≥0.80 to 0.92 [5-8]. Furthermore, studies involving preterm neonates with suspected or confirmed sepsis have consistently shown that higher nSOFA scores are independently associated with increased mortality and significant morbidities, including bronchopulmonary dysplasia and retinopathy of prematurity. Several researches further confirm that nSOFA is a reliable predictor of mortality in very low birth weight infants with late-onset sepsis, supporting its applicability in high-risk preterm populations [6,7].

In recent years, the nSOFA score has emerged as a clinically meaningful tool for evaluating sepsis severity in neonates, particularly compared with traditional scoring systems such as the Score for Neonatal Acute Physiology-II (SNAP-II), Clinical Risk Index for Babies (CRIB-II), and the Töllner Sepsis Score [6,7]. While SNAP-II and CRIB-II are primarily designed for assessing overall illness severity and mortality risk in neonatal intensive care settings, these systems rely heavily on static physiological parameters obtained within a limited time frame and may not adequately capture the dynamic progression of sepsis-related organ dysfunction [5,7]. Similarly, the Töllner Sepsis Score incorporates clinical and laboratory variables but is limited by subjectivity and reduced specificity in preterm populations [5].

In contrast, nSOFA provides an organ dysfunction-focused assessment incorporating respiratory support requirements, cardiovascular instability, and hematologic derangements, which directly reflect the pathophysiological cascade of sepsis. Recent studies (2020-2025) demonstrate that nSOFA has superior prognostic accuracy (AUROC ≥ 0.80-0.90) for mortality prediction than traditional systems, particularly in preterm and very low birth weight infants [5-8]. Moreover, nSOFA allows for serial monitoring, enabling clinicians to track disease progression and response to therapy in real time.

Importantly, nSOFA aligns closely with modern sepsis definitions, emphasizing organ dysfunction rather than isolated inflammatory markers, thereby offering improved clinical relevance [6]. In this patient, the high nSOFA score correlated well with the observed multiorgan failure and fatal outcome, with severe respiratory dysfunction due to bilateral pneumonia, cardiovascular compromise requiring inotropic support, and thrombocytopenia contributing to the overall score. In this case, the likely mechanism of pneumonia was aspiration secondary to impaired swallowing and poor airway protection, compounded by prematurity-related vulnerability, rather than a primary infectious process alone.

While prematurity is a primary contributor to increased susceptibility to infection, Crisponi syndrome may further exacerbate this risk indirectly through impaired swallowing, recurrent aspiration, and autonomic dysfunction. These factors increase the likelihood of respiratory compromise and secondary infections rather than conferring a direct immunological deficit.

Overall, the nSOFA score of 11 correlated with culture-proven severe neonatal sepsis, advanced multiorgan failure, and a rapidly progressive clinical course, consistent with the ultimately fatal outcome observed in this patient.

This case report has several limitations that should be acknowledged. Key perinatal and neonatal parameters were incomplete, including cord blood gas analysis, lactate levels, serial arterial blood gas measurements, and continuous blood pressure trends, which limited objective assessment of perinatal hypoxia and hemodynamic instability. Genetic evaluation was based on WES identifying a homozygous likely pathogenic *CRLF1* variant; however, detailed HGVS-level nomenclature, formal ACMG classification breakdown, and parental segregation studies were not available, limiting deeper genetic interpretation. Although EEG was performed during evaluation of seizure-like episodes and supported a non-epileptic pattern, prolonged video-EEG monitoring and full event correlation were not available, which limits definitive exclusion of intermittent epileptic activity. In addition, microbiological data were partially limited, as repeat blood cultures and complete species-level confirmation with antimicrobial sensitivity profiles were not consistently documented, and the exact timing of antibiotic modifications could not be fully reconstructed. Respiratory support data, while clinically summarized, lacked continuous oxygenation and blood gas-guided titration records, limiting fine-grained analysis of respiratory trajectory. Finally, as a single case report, the findings are inherently limited in generalizability, and causal inferences -- particularly regarding the potential impact of earlier genetic diagnosis on outcome -- should be interpreted with caution.

To our knowledge, this is among the few reported cases of Crisponi syndrome presenting in a preterm neonate complicated by culture-proven gram-negative sepsis due to *Enterobacter* species and severe bilateral pneumonia. Additionally, this case highlights the diagnostic challenge of Crisponi syndrome mimicking HIE in the early neonatal period. The coexistence of prematurity, genetic pathology, and severe infection in this case provides unique clinical insight into the complexity of diagnosis and management in such high-risk neonatal populations.

## Conclusions

This case highlights the diagnostic complexity of Crisponi syndrome in preterm neonates and its potential to mimic HIE. It underscores the increased vulnerability to severe infection due to prematurity and associated complications such as impaired swallowing and aspiration risk. While early genetic diagnosis can facilitate accurate identification and guide management, further studies are needed to better define optimal strategies for improving outcomes in this rare condition. It also showcases the key clinical considerations in the evaluation and management of critically ill neonates. Crisponi syndrome should be included in the differential diagnosis of neonates presenting with dysmorphic features and stimulus-induced spastic episodes, particularly in the setting of consanguinity or unexplained neurological findings. Its neurological manifestations can closely mimic HIE, potentially leading to misdiagnosis and delayed identification of the underlying condition.

Additionally, prematurity combined with impaired swallowing increases the risk of aspiration and severe respiratory infections, predisposing affected neonates to rapid deterioration. In this setting, sepsis may develop quickly and significantly contribute to multiorgan dysfunction and mortality. Early genetic diagnosis, along with prompt multidisciplinary management, is crucial to optimize clinical care, guide targeted interventions, and potentially improve outcomes in similar high-risk neonatal populations.
